# Public Health Information Seeking, Trust, and COVID-19 Prevention Behaviors: Cross-sectional Study

**DOI:** 10.2196/37846

**Published:** 2022-09-30

**Authors:** Emmanuel Kwabena Tetteh, Todd Combs, Elvin Hsing Geng, Virginia Ruth McKay

**Affiliations:** 1 Office of Health Information and Data Science Washington University School of Medicine in St Louis St Louis, MO United States; 2 Center for Public Health Systems Science Washington University in St Louis St Louis, MO United States; 3 Division of Infectious Diseases Washington University School of Medicine in St Louis St Louis, MO United States

**Keywords:** COVID-19, public health, health communication, trust and mistrust, disease prevention, health measure, health information, cross-sectional study, Health Belief Model

## Abstract

**Background:**

Preventative health measures such as shelter in place and mask wearing have been widely encouraged to curb the spread of the COVID-19 disease. People’s attitudes toward preventative behaviors may be dependent on their sources of information and trust in the information.

**Objective:**

The aim of this study was to understand the relationship between trusting in COVID-19 information and preventative behaviors in a racially and politically diverse metropolitan area in the United States.

**Methods:**

We conducted a web-based cross-sectional survey of residents in St. Louis City and County in Missouri. Individuals aged ≥18 years were eligible to participate. Participants were recruited using a convenience sampling approach through social media and email. The Health Belief Model and the Socioecological Model informed instrument development, as well as COVID-19–related questions from the Centers for Disease Control and Prevention. We performed an ordinary least squares linear regression model to estimate social distancing practices, perceptions, and trust in COVID-19 information sources.

**Results:**

Of the 1650 eligible participants, the majority (n=1381, 83.7%) had sought or received COVID-19–related information from a public health agency, the Centers for Disease Control and Prevention, or both. Regression analysis showed a 1% increase in preventative behaviors for every 12% increase in trust in governmental health agencies. At their lowest levels of trust, women were 68% more likely to engage in preventative behaviors than men. Overall, those aged 18-45 years without vulnerable medical conditions were the least likely to engage in preventative behaviors.

**Conclusions:**

Trust in COVID-19 information increases an individual’s likelihood of practicing preventative behaviors. Effective health communication strategies should be used to effectively disseminate health information during disease outbreaks.

## Introduction

As the COVID-19 epidemic continues, preventative behaviors remain an important means to stemming the spread of the infection, despite the availability of effective vaccines and treatment modalities. This need is owed in part to the emergence of new variants of the COVID-19 virus and the low uptake of COVID-19 vaccines globally [1]. A growing body of evidence outlines the importance of nonpharmacological measures, such as restrictions on public gatherings, in controlling and preventing the spread of the disease [2-4]; however, it remains largely variable how willing people are to adopt these behaviors and for how long [5,6].

Initial research outlining the kinds of behaviors that individuals are the most likely to engage in to prevent the spread of COVID-19 is emerging. At the foundation are individuals’ perceptions of risk for acquiring COVID-19 along with its expected severity for each person. Those who perceive themselves to be at a higher risk for acquiring COVID-19 and experiencing a poor outcome from the disease are more likely to engage in preventative behaviors [7-9]. Additionally, the source of information and the trustworthiness of the information is potentially critical. Earlier studies conducted in multiple settings have found that trust in COVID-19 health information from government officials and public health agencies (PHAs) was related to an increase in people’s perceived level of risk, greater severity of the disease if infected, and greater belief in the effectiveness of preventive behaviors [10-17]. Political ideologies, religiosity, and conspiracy ideation have been identified to play a substantial role mediating trust in COVID-19 information and guidelines [13,15], highlighting the demographic differences influencing trust in health information and communication.

This study aimed to contribute to the growing body of evidence targeting the relationship between trust in COVID-19 information and preventative behaviors in St. Louis, Missouri, a relatively small metropolitan area with a racially and politically diverse population, where the burden of COVID-19 was slower to emerge than other major metropolitan areas. This study focused on the early window of the COVID-19 epidemic, prior to the availability of vaccines when state and local officials had imposed policies enacting a number of protective behaviors; however, the majority of the behaviors were voluntary, occurring even before policy measures started going into effect [4]. As of October 5, 2020, there were 32,589 confirmed COVID-19 cases in the St. Louis region including St. Louis City and County. Women had a higher rate of infection (2112 per 100,000) than men (1764 per 100,000). The disease had a 3.2% case fatality rate overall, but there were notable disparities by race—the rate was 2 times higher in Black or African American individuals (169.6 per 100,000) than White individuals (84.4 per 100,000) [18].

## Methods

### Study Design

We conducted a cross-sectional survey from April 23 to July 2, 2020, of St. Louis City and County residents to collect information about perceptions of the COVID-19 epidemic and social distancing behaviors. The survey was administered through Qualtrics and was available via an anonymous link. Participants were not given an incentive for participation; however, for every individual who participated in the survey, a US $1 donation was made to a local nonprofit organization working to counter the economic impacts of the epidemic in the St. Louis region, up to US $2,000. Data were collected from April 23 to July 2, 2020.

### Ethics Approval

The Institutional Review board of Washington University in St. Louis approved the study protocol and procedures of informed consent before the formal survey (#202004131). The Checklist for Reporting Results of Internet E-Surveys was used as a guide to report results and develop this manuscript [19].

### Participants

Individuals aged ≥18 years were eligible to participate in the study. Participants were recruited through targeted social media advertising and distribution through local email listserves. Participant recruitment continued throughout the data collection period. We aimed to collect a representative sample of St. Louis City and County residents based on the following variables: gender, age, socioeconomic status, and race/ethnicity. To increase the representativeness of our sample for St. Louis City and County residents, we constructed sample weights for the regression model. Using 5-year estimates from the American Community Survey (2015-2019) for the public use microdata areas encompassing the city and county [20], we used logistic regression weighting on samples of these data and of the survey data to calculate inverse probability weights. The sample was weighted on household income, race, gender, and age. Due to initially high variance in the weights, we trimmed them according to common practice to produce the final set [21,22].

### Measures

The survey asked participants a series of demographic questions (as shown below) about their zip code of residence, gender, age, ethnicity, race, employment status, social distancing policy, and comorbidities relevant to COVID-19. The survey included questions on comorbid conditions adapted from the Centers for Disease Control and Prevention (CDC) COVID-19 Community Survey Question Bank [23]. Perceptions of the COVID-19 epidemic and social distancing behaviors were measured using individual items corresponding to the major components of the Health Belief Model [24]. The Health Belief Model is a well-established framework that consists of 5 major components: likelihood of action, perceived threat, expected utility, self-efficacy, and cues to action. In the context of adopting preventative COVID-19 behaviors, an individual is likely to engage in social distancing if they perceive themselves to be at risk for COVID-19 (perceived threat), have adequate knowledge of social distancing (cues to action), feel that it will help reduce their risk (expected utility), and feel that they are able to participate in social distancing (self-efficacy) in the context of perceived benefits and barriers of action (expected utility). The survey was also informed by the Socioecological Model, which postulates that health behaviors are affected by factors that occur at individual, interpersonal, community, and societal levels [25]. Participants were asked to select the factors that influence their willingness and ability to engage in preventative behaviors. Lastly, participants were asked about perceptions of how COVID-19 information is communicated, adapted from the Health Information National Trends Survey 4 Cycle 1 instrument [26]. The Health Information National Trends Survey is a well-established, validated instrument that assesses the impact of the health information environment. We adapted questions A7 and A6, using a 4-point Likert scale, to measure participants’ sources of COVID-19 information and trust in COVID-19 information sources, respectively. The full survey instrument is included as an appendix (Multimedia Appendix 1).

### Data Analyses

We downloaded data from Qualtrics and used R statistical software (version 4.0.1; R Foundation for Statistical Computing) for analysis. Descriptive statistical methods were used to summarize data on demographic characteristics. Categorical variables were summarized as frequencies (n) and percentages (%). For the main analyses, we performed an ordinary least squares linear regression model to estimate social distancing knowledge, perceptions, and practices. For the dependent variable, we constructed a preventive behaviors and attitudes (PBA) factor index of 12 reported practices and attitudes toward social distancing and other preventive behaviors, including hand washing, mask wearing, and knowledge and efficacy of social distancing behaviors (see the full list and distributions in Results). This index was operationalized as a proxy for the components of the Health Belief model. We also calculated a trust in public health institutions index from 2 items gauging trust in federal and state and local health agencies to serve as a predictor of social distancing practices and attitudes. The independent variables included this index, demographic characteristics (age, gender, income, race, employment status, and county of residence), and the presence or absence of preexisting health conditions that make individuals more vulnerable to COVID-19. Finally, we included individuals’ perceptions of how likely they were to contract COVID-19 and 2 interaction terms: gender and the level of trust in PHAs; and age and the presence or absence of a preexisting condition that increases COVID-19 vulnerability. We hypothesized from previous literature that women would be more likely than men to take precautions if they had a high level of trust in PHAs [27,28] and that older adults with preexisting conditions would be more likely to take precautions than their younger counterparts [29]. All of these variables and their levels of measurement are described in the beginning of the Results section below.

## Results

### Participant Characteristics

The number of individuals responding to the survey between April 30 and July 2, 2020, was 3180. Among the respondents, 51.9% (n=1650) were aged ≥18 years and lived in St. Louis City or County and thus were eligible for analysis. Participant demographic characteristics are shown in Table 1.

Of the 1650 respondents, just over half (n=879, 53.3%) were aged 18-45 years, and 76.3% (n=1259) were women. Most (n=1426, 86.4%) respondents were White and 96 (5.8%) were Black or African American. More than half (n=912, 55.3%) reported annual household incomes of at least US $70,000, 22.7% (n=375) earned between US $40,000 and US $70,000, 13.6% (n=225) reported incomes less than US $40,000, and 8.4% (n=138) did not respond. About half (805/1650, 48.8%) reported currently working from home, 32.1% (529/1650) were not working, and 18.3% (302/1650) currently worked outside the home.

**Table 1:** Demographics and characteristics.

Characteristic			Respondent (N=1650), n (%)
**Age (years)**			
	18-25	98 (5.9)	
	26-35	368 (22.3)	
	36-45	413 (25)	
	46-55	259 (15.7)	
	56-65	284 (17.2)	
	≥66	227 (13.8)	
**Gender**			
	Gender nonconforming	29 (1.8)	
	Man	346 (21)	
	Woman	1259 (76.3)	
	No response	16 (1)	
**Race/ethnicity**			
	Asian	29 (1.8)	
	Black or African American	96 (5.8)	
	Hispanic or Latino	27 (1.6)	
	Multiple races or ethnicities	53 (3.2)	
	Other	16 (1)	
	White	1426 (86.4)	
	No response	3 (0.2)	
**Household income (US $)**			
	<20,000	62 (3.8)	
	20,000 to <30,000	78 (4.7)	
	30,000 to <40,000	85 (5.2)	
	40,000 to <50,000	130 (7.9)	
	50,000 to <70,000	245 (14.8)	
	70,000 to <100,000	324 (19.6)	
	100,000 to <150,000	312 (18.9)	
	≥150,000	276 (16.7)	
	No response	138 (8.4)	
**Employment status**			
	Working from home	805 (48.8)	
	Not working	529 (32.1)	
	Working outside the home	302 (18.3)	
	No response	14 (0.8)	
**COVID-19–vulnerable health conditions^a^**			
	At least 1	534 (32.3)	
	None	1116 (67.6)	

^a^Respondents were asked about asthma; cancer; chronic heart, kidney, and lung diseases; diabetes; and immunosuppressive conditions.

### Sources of COVID-19 Information

Of the 1650 respondents, most (n=1381, 83.7%) sought out or received information about COVID-19 from PHAs, including local, state, and national PHAs (Table 2). In all, 58.7% (n=969) of respondents had sought or received information from both a local or state health department and the CDC, 3.4% (n=56) had done so only from state or local PHAs, 21.6% (n=356) had gotten information solely from the CDC, and 16.3% (n=269) had not received any information from a PHA. Most respondents had a moderate (n=751, 45.5%) or high (n=512, 31%) amount of trust in federal PHAs, and the remaining 23.2% (n=382) had little or no trust in federal PHAs (Table 2). Similarly, for local or state PHAs, most had a moderate (n=801, 48.5%) or high (n=495, 30%) amount of trust, and 21% (n=347) had little or no trust.

**Table 2:** Sources of information, trust in public health agencies, and perceptions of risk.

Topic			Respondent (N=1650), n (%)
**Sources of information**			
	None	269 (16.3)	
	State or local PHA^a^	56 (3.4)	
	CDC^b^	356 (21.6)	
	State or local PHA and CDC	969 (58.7)	
**Trust in federal PHAs**			
	Not at all	100 (6.1)	
	A little	282 (17.1)	
	A moderate amount	751 (45.5)	
	A lot	512 (31)	
	No response	5 (0.3)	
**Trust in state or local PHAs**			
	Not at all	61 (3.7)	
	A little	286 (17.3)	
	A moderate amount	801 (48.5)	
	A lot	495 (30)	
	No response	7 (0.4)	
**Likelihood of contracting COVID-19**			
	Unlikely	626 (37.9)	
	Neither	498 (30.2)	
	Likely	517 (31.3)	

^a^PHA: public health agency.

^b^CDC: Centers for Disease Control and Prevention.

### Perceptions of Risk and Testing

Table 2 also shows the perceptions of risk in the population. Of the 1650 respondents, about one-third (n=517, 31.3%) of participants thought that they were likely to contract COVID-19 in the next 3 months, and a larger percentage (n=626, 37.9%) responded that they were unlikely to contract COVID-19 in the next 3 months. The remaining 30.2% (n=498) responded that they were neither likely nor unlikely. About one-third (n=534, 32.3%) of respondents reported preexisting health conditions that made them more likely to contract or experience moderate or severe cases of COVID-19 (eg, asthma and chronic heart, kidney, or lung disease).

### Preventive Behaviors and Social Distancing

**Figure 1:**
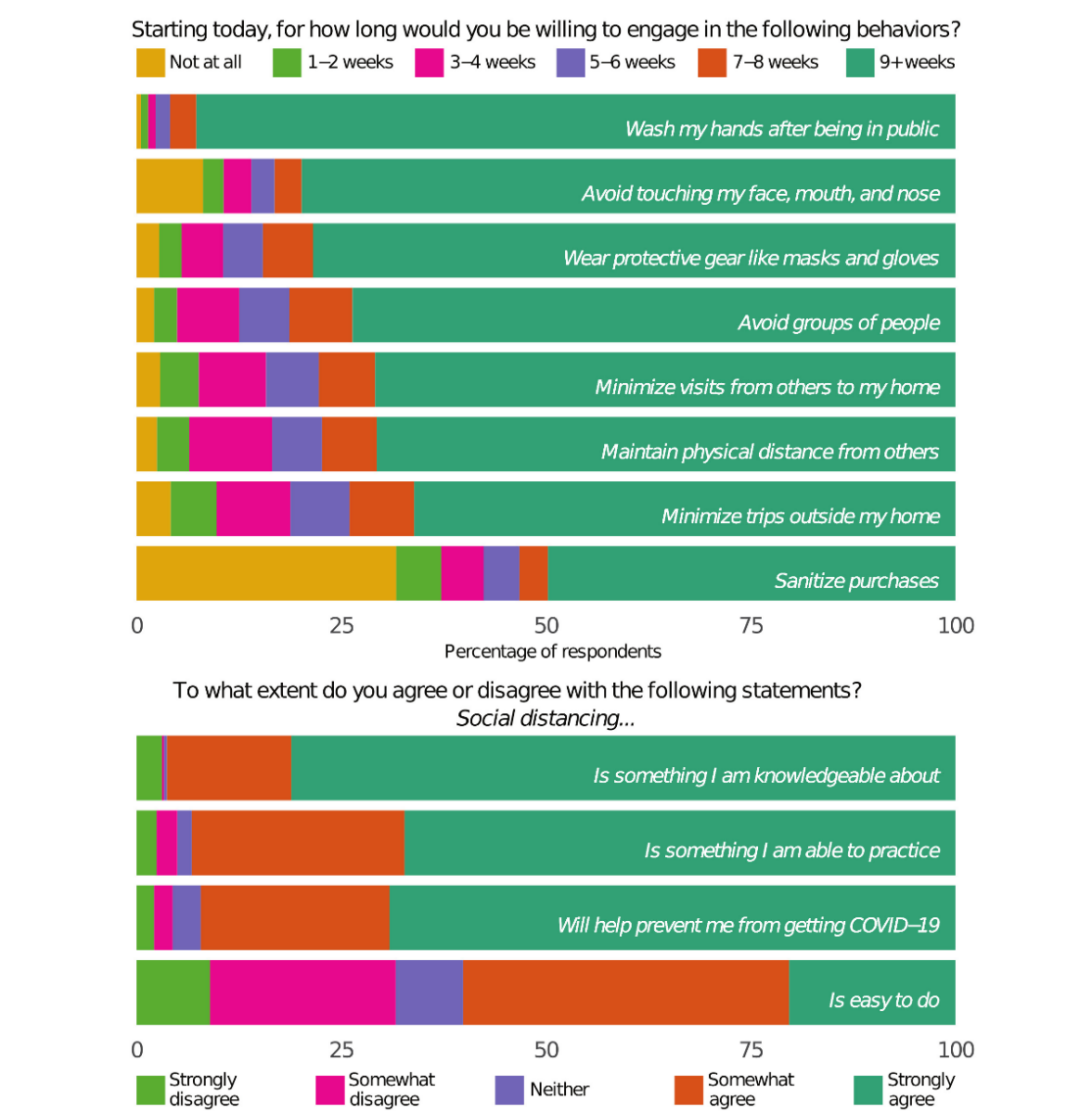
Willingness to practice and attitudes toward preventative behaviors.

The survey also asked about which social distancing and other preventive behaviors the respondents were engaging in and how long they were willing to do so (Figure 1). Of the 1650 respondents, the clear majority—at least 75%—were willing to engage in 4 of the 8 specific behaviors for 9 weeks or more: washing hands after being in public (n=1512, 91.6%), avoiding touching one’s face (n=1298, 78.7%), wearing protective gear (n=1282, 77.7%), and avoiding groups (n=1203, 72.9%). Between half and three-quarters of the respondents were willing to engage in the other 4 behaviors for 9 weeks or more: minimizing in-home visitors (n=1155, 70.9%) and trips from home (n=1080, 65.5%), maintaining physical distance from others (n=1161, 70.4%), and sanitizing purchased goods (n=797, 46.5%). The majority of respondents either strongly (n=1331, 80.7%) or somewhat (n=249, 15.1%) agreed with the statement “I am knowledgeable about social distancing.” Similarly, 66.5% (n=1098) strongly agreed and 25.7% (n=424) somewhat agreed that they were able to practice social distancing, and a comparable number of respondents agreed somewhat (n=377, 22.8%) or strongly (n=1129, 68.4%) that social distancing would help prevent COVID-19 transmission. However, when asked whether social distancing was easy to do, only 20.2% (n=333) strongly and 39.6% (n=653) somewhat agreed, with 22.5% (n=372) somewhat and 8.9% (n=147) strongly disagreeing with the statement.

### Linear Regression

**Figure 2:**
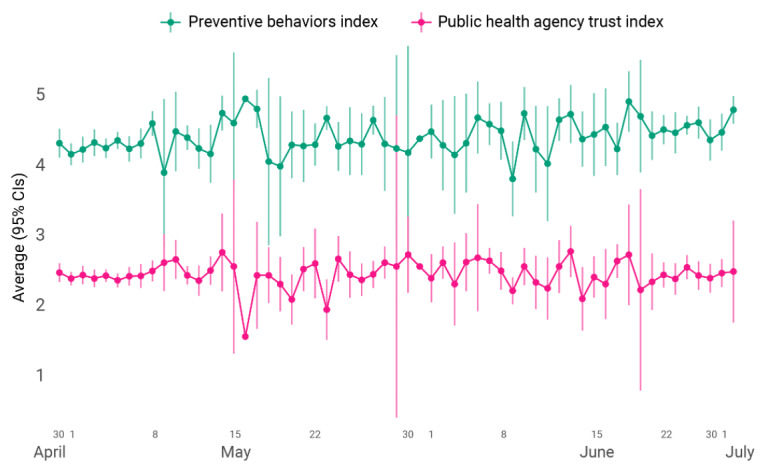
Average preventative behaviors and public health trust indices throughout data collection: April 30 to July 2.

To model whether respondents’ actions and attitudes were influenced by demographics, sources of COVID-19 information, trust in those sources, and perceptions of risk for getting COVID-19, we constructed a factor index of the 12 survey items in Figure 1. The PBA index had a Cronbach α of .83 (95% CI .81-.84) and ranged from 0.41 to 5. The mean value was 4.2 (SD 0.82), and the natural log of this index was used as the outcome variable to approximate linearity. A higher score on the index means more practice of preventative behaviors. We also constructed a trust in public institutions index from the 2 survey items on trust in information from local or state and federal PHAs to serve as a predictor variable. The trust index had a Cronbach α of .80 (95% CI .79-.82) and ranged from 0 to 3, with a mean of 2.0 (SD 0.75). A higher score on the trust index means more trust in a PHA. From April 30 to July 2, 2020, both of these indexes stayed relatively constant (Figure 2), with daily reported averages around the overall mean for each.

Table 3 presents the results of the linear regression model. As explained above in the Methods section, the model used inverse probability weights to increase the representativeness of the populations of St. Louis City and County. Although the variance of the initial weights was relatively high (0.4; range 3.0-5.5), after trimming, it was 0.27 (range 3.0-5.2). The outcome of the model—the (natural log of the) PBA index—was regressed on 3 main categories of variables: demographics, trust in public health institutions, and individual risk perceptions. In addition, 2 interaction terms were included as moderators, and a control variable for time gauged the evolution of preventive behaviors throughout the 10 weeks of data collection. We executed the model in R statistical software (version 4.0.1) using the *svyglm* function in the *survey* package [30], which calculates robust standard errors to account for the weights included in the model.

**Table 3:** Linear regression results: the effect of demographic characteristics, risk perception, and public health agency trust on COVID-19 preventative attitudes and behaviors (N=1440; adjusted *R^2^*=0.12). Outcome is the natural log of the factor index of willingness to and attitudes toward preventive behaviors. CIs were calculated with heteroskedasticity-robust standard errors. When checked for multicollinearity, the maximum variance inflation factor value was 1.09 for the age predictor.

Variable					OLS^a^ coefficient			95% CI		*P* value
**Demographics**										
	**Age (years)**									
		18-45	Reference			Reference				
		46-65	0.03			0.00 to 0.06			.05	
		≥66	0.05			0.00 to 0.09			.10	
	**Gender**									
		Women	Reference			Reference				
		Men	–0.68			–1.06 to –0.29			<.001	
	**Income (US $)**									
		<40,000	Reference			Reference				
		40,000 to <70,000	0.04			0.00 to 0.08			.05	
		≥70,000	0.02			–0.00 to 0.06			.16	
	**Race/ethnicity**									
		Black or African American	–0.01			–0.05 to 0.04			.85	
		Other races/ethnicities	0			–0.05 to 0.06			>.99	
		White	Reference			Reference				
	**Employment status**									
		Working outside the home	Reference			Reference				
		Working from home	0.05			0.02 to 0.08			<.001	
		Not working	0.05			0.01 to 0.08			.01	
	**County of residence**									
		St. Louis City	Reference			Reference				
		St. Louis County	–0.02			–0.05 to –0.00			.04	
	**Vulnerable conditions**									
		None	Reference			Reference				
		At least 1	0.05			0.02 to 0.08			.01	
**Public health institutions**										
	Trust in PHAs^b^ (index, log-transformed)			0.12			0.02 to 0.22			.03
	Trust in doctors			0.03			–0.01 to 0.06			<.001
	Amount of evidence from PHAs			0.01			0.00 to 0.03			.10
**Likelihood of getting COVID-19**										
	**Individual perception**									
		Not likely	Reference			Reference				
		Neither	0.01			–0.03 to 0.03			.96	
		Likely	0.02			–0.01 to 0.05			.11	
**Interaction terms**										
	**Gender and trust in PHAs**									
		Women	Reference			Reference				
		Men	0.52			0.22 to 0.82			<.001	
	**Age (years) and vulnerable conditions**									
		18-45	Reference			Reference				
		46-65	–0.06			–0.11 to 0.00			.05	
		≥66	–0.05			–0.12 to 0.02			.24	
**Time**										
	Week number (April 30 to July 2)			0.02			0.00 to 0.01			<.001

^a^OLS: ordinary least squares.

^b^PHA: public health agency.

Since the dependent variable was log-transformed to better approximate linearity, the interpretation of the coefficients requires an extra step. For all but trust in PHAs index, which was also log-transformed, the formula for back-transforming the coefficient estimates is as follows:




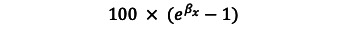




where *β_x_* is the estimated coefficient. Once transformed using this formula, the coefficients are approximately multiplied by 100.

For demographic characteristics, one difference was found between the 3 age groups. Those aged 46-65 years scored 3% higher on the PBA index than their younger (aged 18-45 years) counterparts (*P*=.05), all else equal. Women in our sample were estimated to have PBA index scores two-thirds (68%) higher than men on average (*P*<.001), and scores for middle-income individuals reporting between US $40,000 and US $70,000 in annual household income were 4% higher than those reporting less than US $40,000 (*P*=.05). No statistically significant differences were found between individuals who were White, Black or African American (*P*=.85), and of other races or ethnicities (*P*>.99). Respondents who were not working and those who were working from home both scored 5% higher than those working outside the home for preventive behaviors (*P*<.001 and *P*=.03, respectively). Residents of St. Louis County scored 2% lower than those in the city on average (*P*=.04), and those with at least 1 COVID-19–vulnerable health condition reported 5% higher social distancing attitudes and practices than those with no related conditions (*P*=.01).

The relationship between trust in PHAs and preventive behaviors was elastic, since both the independent and dependent variables were log-transformed, and can be interpreted as for every 12% increase in trust, a 1% increase in precautionary practices resulted. Trust in PHAs also moderated the relationship between respondents’ gender and preventive behaviors and attitudes, as increased trust among men narrowed the gap between the genders by 0.52% as further illustrated below (*P*<.001). To a lesser extent, the presence of vulnerable conditions led to decreased differences (–6%) between respondents aged 46-65 and 18-45 years in preventive behaviors (*P*=.05). Trust in doctors, the amount of COVID-19 evidence received from PHAs, and individual risk perceptions were not related to preventive behaviors after controlling for the effects of all other variables in the model. Finally, the positive and statistically significant effect of the variable for week completing the survey suggested a slight 2% increase on average over time (*P*<.001).

**Figure 3:**
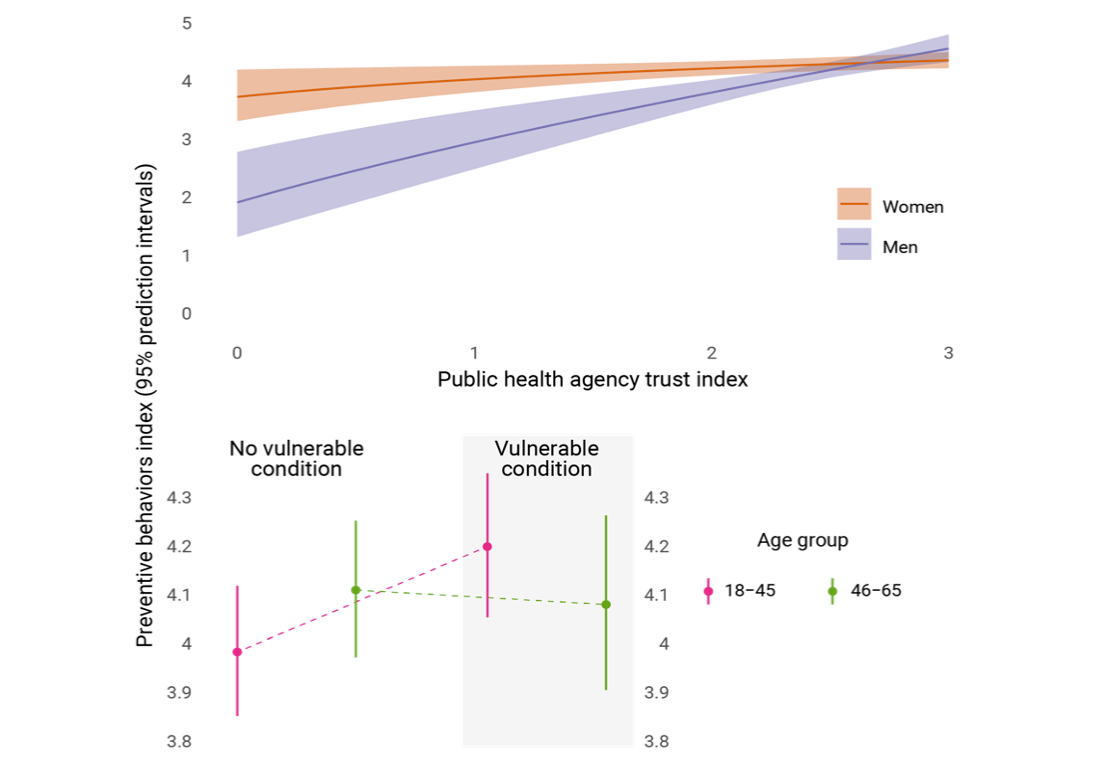
Model estimates for practicing behaviors (scale: 0=low to 5=high) for interaction terms (top: gender and public health trust; bottom: age and vulnerable health conditions). Results were calculated using the average values of all other covariates.

To further illustrate how trust in PHAs led to decreased differences across gender, the top panel in Figure 3 shows the average predictions for women and men along the range of trust. Although the largest differences in preventive behaviors and attitudes is present at the lowest levels of trust—women at about 4 on the index versus men at 2.7, representing a 67% difference—as trust in PHAs increases among men, the differences narrow and eventually disappear at the highest trust levels. The bottom panel of Figure 3 considers the impact of preexisting COVID-19–vulnerable conditions on the differences between those aged 18-45 and 46-65 years for preventive behaviors. For those with no such conditions, the younger group is about 6% lower on the index. Those aged 18-45 years with vulnerable conditions have preventive behaviors and attitudes 5% higher than the same group without conditions and are 3% higher on the index than their older counterparts who also have vulnerable conditions. The relative scores for preventive behaviors flip between the 2 age groups when comparing those with and without vulnerable conditions.

## Discussion

### Principal Findings

The purpose of this study was to investigate the relationship between trust in COVID-19 information and engaging in preventive behaviors among residents in the St. Louis region, including St. Louis City and County, in the early window of the epidemic prior to the development of vaccines and treatment modalities. The majority of respondents had sought or received COVID-19–related information from a PHA and trusted that information. Those who expressed trust in the information from PHAs were more likely to engage in preventative behaviors. Our results show that PHAs are still an important source of information in disease outbreaks, and contrary to the vocalization of people not obeying [31,32], the majority of people still listen to their PHAs. Across all demographic groups, preventative behaviors improved as trust increased. In our sample, people’s trust in sources of information and their practice of preventative behaviors remained relatively consistent with a slight 2% increase on average throughout the period of data collection, regardless of the changes in the severity of the disease (ie, caseload and case fatality rate) in the region.

This study contributes to the limited scientific literature regarding the association between COVID-19 preventative behaviors, the trustworthiness of information, and sources of information. Our findings are comparable to earlier studies that found that people who had higher trust in government COVID-19 messaging were more likely to adopt preventive behaviors [7,10-12,14,15]. In alignment with existing work, our respondents felt that preventive measures, such as social distancing, would help prevent the disease spread, but only a minority of people strongly agreed that social distancing was easy to do [33]. Public health campaigns are usually implemented under the assumption that once information is disseminated and knowledge is enhanced, recommended behaviors will follow [34]. Our findings demonstrate that this assumption is not always valid; rather, people’s perception of risk and their ability to engage in preventive behaviors (self-efficacy) are more likely to influence their health behavior [35,36].

A potential threat to people’s accurate perception of COVID-19 risk is through misinformation. Although it is unclear to what extent misinformation among our study population impacted our results, other studies have reported the prevalence of widespread false information about the COVID-19 disease and the effect of misinformation on people’s perceived risk and adoption of preventative behaviors [37,38]. Results from a study conducted in 52 countries showed that 83% of vaccine-related rumors on popular web-based platforms were false [38], posing a substantial threat to vaccine uptake. It is incumbent upon PHAs, clinicians, and health practitioners to ensure that the most accurate and up-to-date disease risk information and preventative measures are carefully distilled and communicated to the public. Furthermore, it is imperative for public health messaging to debunk misleading and false information about the disease, modes of transmission, and the effectiveness of treatment and preventative measures.

Additionally, our study highlights a need for audience-targeted health communication that can effectively encourage different groups of people within a given population—specifically young people, men, and lower-income populations—to increase trust in the health information provided. Segmenting audience according to various demographic characteristics and behavioral traits increases the effectiveness of health communication campaigns intended to promote health behaviors [39]. The Risk Perception Attitude framework—a tool for assessing individuals’ health behaviors based on their perceived risk and efficacy—can be used to guide health communication to different groups of people [40].

### Limitations

This study used a cross-sectional study design, which potentially limits the generalizability and representativeness of the results. The study population was not representative of the actual population, with some demographic groups substantially underrepresented, therefore reducing the generalizability of the results. This limitation was addressed by constructing sample weights for the regression model. The sample was weighted on household income, race, gender, and age, and our large sample size helped ensure statistical power. Since this study recruited participants voluntarily, people who were concerned by the COVID-19 disease or had been affected by it may have been more likely to participate, thus introducing a possible selection bias. Lastly, the survey responses were self-reported and may have led to some recall bias.

### Conclusion

This study provided insights into how preventive behaviors during the COVID-19 epidemic are influenced by sources of health-related information and the trustworthiness of information. We found that contrary to the vocalization of people not obeying, the majority of people still listen to their PHAs. Receiving health information from PHAs—and trusting that information—increased an individual’s likelihood of engaging in preventative health behaviors. Incoherent COVID-19 information from state and local PHAs and blanket approaches to communicating health information have a decreased impact in addressing risk perceptions and efficacy beliefs in specific subpopulations, such as among men and young adults. Future research should consider how audience-targeted health communication strategies can ensure that different subpopulations adopt preventive health behaviors in disease outbreaks. Furthermore, PHAs and clinicians should make a continuous effort to debunk false and misleading COVID-19 information that may be prevalent on the internet and social media.

## Appendix

Multimedia Appendix 1 The full COVID-19 survey instrument.

## Abbreviations

CDC: Centers for Disease Control and Prevention
PBA: preventive behaviors and attitudes
PHA: public health agency
