# Supplementary material for: Public Health Information Seeking, Trust, and COVID-19 Prevention Behaviors: Cross-sectional Study
Source: J Med Internet Res. 2022 Sep 30;24(9):e37846. doi: 10.2196/37846 (PMC9528929; doi:10.2196/37846)
Supplement: Multimedia Appendix 1 [file jmir_v24i9e37846_app1.pdf]

COVID-19 Instrument

Start of Block: Information Sheet

Q1
We invite you to participate in a research study being conducted by investigators from Washington University in St. Louis to understand your opinions and behaviors related to the COVID-19 epidemic.
Your responses will be used to inform COVID-19 and social distancing efforts locally. You are free to skip any questions you do not feel comfortable answering.

If you have questions for the research team, please contact Dr. Virginia McKay, virginia.mckay@wustl.edu or you may contact the Human Research Protection Office at 1-(800)-438-0445 or hrpo@wustl.edu.

Thank you very much for your consideration of this research study.

Page Break

End of Block: Information Sheet

Start of Block: Screening

Q2 In order to make sure that you meet our criteria and that you are the right person to participate in this survey, please answer the question below.

Q3 Are you 18 years or older?

- No, I am not (15)
- Yes, I am (16)

Skip To: End of Block If Are you 18 years or older? = Yes, I am

Skip To: Q4 If Are you 18 years or older? = No, I am not

Page Break

Q4 We apologize, but you are not eligible to participate in our survey at this time.
Thank you for your consideration of this research study

Skip To: End of Survey If We apologize, but you are not eligible to participate in our survey at this time. Thank you for... Is Displayed

Page Break

End of Block: Screening

Start of Block: SECTION 1. Participant Characteristics

Q5
This first set of questions will ask about you and your background.

Q6 How old are you?

○ 18-25 (1)

○ 26-35 (2)

○ 36-45 (3)

○ 46-55 (4)

○ 56-65 (5)

○ 66-75 (6)

○ 76-85 (7)

○ 86 or older (8)

---

*

Q7 What is your 5-digit zip code where you live?

____

Q8 Do you live in St. Louis city or county, or somewhere else?

- St. Louis City (1)
- St. Louis County (2)
- St. Charles County (6)
- Jefferson County (7)
- Another county or place in Missouri (3)
- Another county or place in Illinois (4)
- Other (5)

Q9 What is your ethnicity or race? (Please select all that apply)

- American Indian or Alaska Native (1)
- Asian (2)
- Black or African American (3)
- Hispanic or Latino (7)
- Native Hawaiian or other Pacific Islander (4)
- White (5)
- Other (6)

Q10 How do you describe yourself?

○ A man (1)

○ A woman (2)

○ Gender nonconforming / Other (3)

○ Prefer not to respond (4)

Q11 Including yourself, how many people live in your household?

▼ 1 (2) ... 10 or more (12)

Q12 Of the people that live in your household, how many are under 18?

▼ 0 (2) ... 10 or more (13)

Q13 Of the people that live in your household, how many are over 65?

▼ 0 (1) ... 10 or more (11)

Q14 Which of the following categories best describes your ANNUAL household income? Annual household income is the income before taxes of *all persons* in your household *combined* for *one year*?

- ○ Less than $20,000 (1)
- ○ $20,000 to less than $30,000 (2)
- ○ $30,000 to less than $40,000 (3)
- ○ $40,000 to less than $50,000 (4)
- ○ $50,000 to less than $70,000 (5)
- ○ $70,000 to less than $100,000 (6)
- ○ $100,000 to less than $150,000 (7)
- ○ $150,000 or more (8)
- ○ Prefer not to respond (9)
- ○ I do not know (10)

Page Break

End of Block: SECTION 1. Participant Characteristics

Start of Block: SECTION 1a.

Q15 Do you have any of the following conditions? (Please select all that apply)

- ☐ Asthma (1)
- ☐ Cancer in the past year (2)
- ☐ Chronic heart disease (High blood pressure, a recent heart attack, etc.) (3)
- ☐ Chronic kidney disease (4)
- ☐ Chronic lung disease (COPD, emphysema, etc.) (6)
- ☐ Diabetes (High sugar disease) (7)
- ☐ Immunosupressive condition (Lupus, HIV, etc.) (8)
- ☐ ⊗None of the above (9)

Q16 What type of health insurance do you currently have?

☐ No insurance/Self-pay (1)

☐ Employer-sponsored disability insurance (2)

☐ Medicaid (3)

☐ Medicare (4)

☐ National Health Insurance (5)

☐ Private or group health insurance (6)

☐ Veterans Affairs/Military (7)

☐ Unknown (8)

☐ Other, please specify (9) ____

Q17 Are you living in an area with a policy mandating social distancing measures (e.g., shelter-in-place or safer-at-home orders)?

○ No (3)

○ Yes (4)

○ I don't know (5)

Q18 Are you currently employed?

- No (11)
- Yes (12)
- Yes, but I've been furloughed (13)

Skip To: Q19 If Are you currently employed? = Yes

Q21 Were you furloughed or laid off because of your employer's response to the COVID-19 (the coronavirus) epidemic?

- No (1)
- Yes (2)

Display This Question:

If Are you currently employed? = Yes

Q19 Are you currently working from home?

- No, I'm still working in my office or business (1)
- Yes, I'm working from home (2)

Display This Question:

If Are you currently employed? = Yes

Q20 For your work, do you have to interact with customers face to face?

- No (1)
- Yes (2)

Q49 Has your employer provided any of the following COVID-related resources for you? (Select all that apply)

- ☐ ⊗I do not receive any resources from my employer (5)
- ☐ Masks (1)
- ☐ Information on COVID-19 (2)
- ☐ Support services like counseling or an emergency fund (3)
- ☐ Other (4) ____
- ☐ ⊗Does not apply to me (6)

Page Break

End of Block: SECTION 1a.

Start of Block: SECTION 2. Perceptions of COVID-19

Q22 **The following questions will ask about your perceptions of COVID-19 (the coronavirus) and social distancing.**

Q23 In the past 30 days, have you had any of the following symptoms? (Please select as many as apply)

- ☐ Fever (1)
- ☐ Dry Cough (2)
- ☐ New or worsened shortness of breath (3)
- ☐ Runny nose (4)
- ☐ Itchy/watery eyes (5)
- ☐ Diarrhea or vomiting (6)
- ☐ Loss of sense of smell or taste (7)
- ☐ Intense fatigue (8)
- ☐ Body aches (9)
- ☐ I have not had any of these symptoms (10)

Q24 Have you had any symptoms that made you believe you had COVID-19 (including the ones checked above or others)?

- Yes (1)
- No (2)

Q25 Have you been tested for COVID-19?

- Yes (1)
- No (2)

Q26 Have you been diagnosed with COVID-19?

- Yes (1)
- No (2)

Display This Question:

If Have you been tested for COVID-19? = No

Q27 Would you like to be tested for COVID-19?

- Yes (1)
- No (2)

Q28 Within the next three months, how likely do you think you are to...

	Extremely unlikely (1)	Somewhat unlikely (2)	Neither likely nor unlikely (3)	Somewhat likely (4)	Extremely likely (5)
get COVID-19? (1)	○	○	○	○	○
be hospitalized because of COVID-19? (2)	○	○	○	○	○
practice social distancing? (3)	○	○	○	○	○

Q29 To what extent do you agree or disagree with the following statements?

	Strongly disagree (1)	Somewhat disagree (2)	Neither agree nor disagree (3)	Somewhat agree (4)	Strongly agree (5)
I am knowledgeable about social distancing (1)	○	○	○	○	○
I am able to practice social distancing (2)	○	○	○	○	○
Social distancing will help prevent me from getting COVID-19 (3)	○	○	○	○	○
Social distancing is easy to do (4)	○	○	○	○	○

Page Break

End of Block: SECTION 2. Perceptions of COVID-19

Start of Block: SECTION 4 Qualitative Questions

Q30 In one or more sentences, please describe how the COVID-19 epidemic has impacted you.

____

____

____

____

____

Page Break

End of Block: SECTION 4 Qualitative Questions

Start of Block: SECTION 3. Factors influencing Social Distancing

Q31 **We would like to know more about how you practice social distancing**.

By social distancing we mean deliberate actions to minimize contact with other people and reduce the spread of COVID-19 (the coronavirus).

Q32 What do you do when practicing social distancing? (Please select all that apply)

- ▢ I maintain a physical distance from other individuals (1)
- ▢ I avoid groups of people (2)
- ▢ I minimize trips requiring me to leave my home (3)
- ▢ I minimize visits from other people to my home (4)
- ▢ I wear protective gear like masks and gloves (5)
- ▢ I wash my hands after being in public (6)
- ▢ I sanitize purchased goods (7)
- ▢ I avoid touching my face, mouth, and nose (8)
- ▢ None of the above (9)
- ▢ Other (please specify) (10) ____

Q33 Starting today, for how long would you be willing to engage in the following behaviors?

	Not at all (1)	1-2 weeks (2)	3-4 weeks (3)	5-6 weeks (4)	7-8 weeks (5)	9 or more weeks (6)
Maintain a physical distance from other individuals (1)	o	o	o	o	o	o
Avoid groups of people (2)	o	o	o	o	o	o
Minimize trips requiring you to leave your home (3)	o	o	o	o	o	o
Minimize visits from other people to your home (4)	o	o	o	o	o	o
Wear protective gear like masks and gloves (5)	o	o	o	o	o	o
Wash your hands after being in public (6)	o	o	o	o	o	o
Sanitize purchased goods (7)	o	o	o	o	o	o
Avoid touching your face, mouth, and nose (8)	o	o	o	o	o	o

End of Block: SECTION 3. Factors influencing Social Distancing

Start of Block: Section 4 Health Communication

Q40 **This last section will ask questions about your perceptions of how COVID-19 information is communicated.**

Q41 In general, how much would you trust information about health or medical topics about COVID-19 from each of the following sources?

	Not at all (1)	A little (2)	A moderate amount (3)	A lot (4)
A doctor (1)	○	○	○	○
Family or friends (2)	○	○	○	○
Federal government health agencies (3)	○	○	○	○
State or local government health agencies (8)	○	○	○	○
Community organizations (4)	○	○	○	○
National political leaders (9)	○	○	○	○
State or local political leaders (10)	○	○	○	○
Religious organizations & leaders (5)	○	○	○	○
Internet Sources (6)	○	○	○	○
Social Media (7)	○	○	○	○

Q42 Imagine that you had a strong need to get information about COVID-19. Where would you go first?

- ○ Books (1)
- ○ Brochures, pamphlets, etc. (2)
- ○ Complementary, alternative, or unconventional practitioner (3)
- ○ Doctor or health care provider (4)
- ○ Family (5)
- ○ Friend/Co-worker (6)
- ○ Internet (7)
- ○ Library (8)
- ○ Magazines (9)
- ○ Newspapers (10)
- ○ Social Media (11)
- ○ Telephone information number (12)
- ○ Other (please specify) (13) ____

Q50 Have you seen, received, or sought information from...

	Yes (1)	No (2)
A City Health Department (1)	○	○
A County Health Department (2)	○	○
A State Health Department (3)	○	○
The CDC (Centers for Disease Control and Prevention) (4)	○	○

End of Block: Section 4 Health Communication

Start of Block: End of Survey

Q43 Thank you for completing the survey. We value your input as a way to inform our response to the COVID-19 epidemic.

If you have any questions or concerns, please don't hesitate to contact Dr. Virginia McKay by email at virginia.mckay@wustl.edu

*

Q44
If you are interested in participating in future work related to COVID-19, please leave your email address or telephone number below.

○ Email address (1) ____

○ Email address (for verification) (2) ____

○ Phone number (XXX) XXX-XXXX (3) ____

End of Block: End of Survey
